# Pulmonary pathology in white-eared opossums (*Didelphis albiventris*) with natural *Heterostrongylus heterostrongylus* (Travassos, 1925) infections

**DOI:** 10.1590/S1984-29612025022

**Published:** 2025-05-19

**Authors:** Maria Izabel Pedra Sogari, Andressa Maria Rorato Nascimento de Matos, Felipe Martins Negreiros Navolar, Amanda Filus Marchese, Jorgeana Guadanhini Negrizolli, Fernando de Souza Rodrigues, Ana Paula Frederico Loureiro Bracarense

**Affiliations:** 1 Departamento de Medicina Veterinária Preventiva, Universidade Estadual de Londrina – UEL, Londrina, PR, Brasil

**Keywords:** Histopathology, respiratory system, opossum, vascular changes, lungworms, Histopatologia, sistema respiratório, gambás, alterações vasculares, parasitas pulmonares

## Abstract

Carcasses of road-killed wild animals provide critical insights into animal health and environmental conditions. However, data on the presence of lung parasites and their effects on the health of white-eared opossums are limited. This study examines lung parasites and their pathological effects on white-eared opossums (*Didelphis albiventris*) found in northern Paraná, Brazil. We collected and processed lung samples from 21 opossums using standard histological techniques. Lung nematodes were preserved and identified by their morphological traits. *Heterostrongylus heterostrongylus* (Travassos, 1925) was detected in nine animals (42.8%). Key histological observations included diffuse interstitial pneumonia, lymphoid tissue hyperplasia, pulmonary hemorrhage, smooth muscle hypertrophy in the pulmonary vessels, congestion, and edema. Although the immediate cause of death was hypovolemic or neurogenic shock from vehicular collisions, the findings underscore the significance of studying road-killed wild animals to evaluate wildlife health and environmental status. This research also documents the first instance of *H. heterostrongylus* lung infections in *D. albiventris*, suggesting that these parasites significantly impact lung health.

## Resumo

Carcaças de animais silvestres atropelados fornecem informações valiosas sobre a saúde animal e as condições ambientais. Este estudo teve como objetivo investigar a ocorrência de parasitas pulmonares e seus efeitos patológicos no sistema respiratório de gambás-de-orelha-branca (*Didelphis albiventris*), atropelados na região norte do Paraná, Brasil. Amostras de pulmão de 21 gambás foram colhidas e processadas de acordo com protocolos histológicos padrão. Os nematódeos pulmonares foram fixados e identificados com base nas características morfológicas. *Heterostrongylus heterostrongylus* (Travassos, 1925) foi identificado em nove animais (42,8%). Os principais achados histológicos incluíram pneumonia intersticial difusa, hiperplasia do tecido linfoide, hemorragia pulmonar, hipertrofia da camada muscular de vasos pulmonares, congestão e edema pulmonar. Apesar desses achados, todas as mortes foram atribuídas a choque hipovolêmico ou neurogênico, causado por traumas decorrentes de colisões com veículos. Este estudo destaca a importância de utilizar animais silvestres atropelados para avaliar sua saúde e o estado do ambiente em que vivem. Adicionalmente, relata pela primeira vez a infecção pulmonar por *H. heterostrongylus* em *D. albiventris*. As evidências histológicas sugerem que o parasito contribui para lesões pulmonares que podem comprometer a saúde dos gambás.

## Introduction

Urban sprawl and road construction have dramatically increased roadkill incidents worldwide, affecting species ranging from small amphibians to large mammals and severely impacting local populations (Farhig & Rytwinski, 2009). In Brazil, roadkill significantly contributes to local biodiversity loss, with estimated 475 million wild animals killed annually (UFLA, 2016).

Road-killed animals carcasses provide crucial data for scientific research and wildlife conservation. Utilizing these carcasses in epidemiological studies offers valuable insights into parasite-host interaction and helps identify environmental reservoirs (Richini-Pereira et al., 2014). According to the Brazilian Center for Road Ecology Studies (CBEE), among the 56 species of Brazilian marsupials (Cáceres, 2013), the white-eared opossum *(Didelphis albiventris*), is notably vulnerable to vehicle collisions due to its adaptation to urban and peri-urban environments. This species is abundant and significant in public health, serving as a reservoir for various zoonoses (Lima et al., 2012; Xavier et al., 2014). Despite numerous studies on its zoonotic potential, little is known about the helminthic community in marsupials (Jiménez et al., 2011; Paiz et al., 2016; Fornazari et al., 2018).

Helminth infections are known to debilitate wild mammal populations significantly (Lamberski et al., 2002). However, specific data on the impact of the Angiostrongylidae family, which include respiratory parasites, are sparse. Notably, *Didelphostrongylus hayesi* has been reported in the lungs of several opossum species (*D. virginiana* and *D. marsupialis*) (Prestwood, 1976; Duncan et al., 1989; Monet-Mendoza et al., 2005), and *Heterostrongylus heterostrongylus* (Travassos, 1925) has been identified in *D. aurita* (Travassos, 1925; Costa et al., 2016). Given the limited literature, this study assesses the presence of lung nematodes and their respiratory effects on *D. albiventris* found road-killed in the northern Paraná, Brazil.

## Material and Methods

Autopsies were conducted on 40 *D. albiventris* specimens that had been run over on highways in the north of Parana, Brazil, from January 2017 to September 2023 in the Central-North Mesoregion of Parana, Southern Brazil. Of these, tissues from 21 animals were submitted to histological analysis. Eighty-five percent of the evaluated animals (18/21) were recorded within the municipality of Londrina (23°18'16.2”S, 51°10'10.6”W), while three individuals were found along highways in adjacent municipalities (Tamarana 23°21'32”S, 51°15'44”W, and São Jerônimo da Serra 23°22'10”S, 51°30'10”W). In Londrina, the animals were predominantly observed in urban areas adjacent to riparian zones, characterized by forested corridors that border small streams and extend throughout the municipality.

Only specimens in fresh or moderately decomposed states (Pugliares et al., 2007) underwent standard macroscopic and subsequent histopathological evaluations. Lung fragments were fixed in 10% buffered formalin solution and processed using routine histological methods. Briefly, the samples were dehydrated in increasing concentrations of alcohol, cleared in xylene, embedded in paraffin, and stained with hematoxylin and eosin (Feldman & Wolfe, 2014). Masson's Trichrome stain was employed to assess vascular changes and tissue fibrosis (Sannad et al., 2017).

Nematodes extracted from the lungs were preserved in 70% ethanol. Identification relied on foundational descriptions by Anderson et al. (1980) and Costa et al. (2016). Due to the parasites` fragility, clarification techniques were not feasible. Both male and female nematodes were identified under a 40x magnification using an Olympus BX43 optical microscope equipped with Q-Color3™ Imaging System (Olympus Corporation, Tokyo, Japan) for photographic documentation. The images were analyzed using CellSens Standard software, version 1.15 2016 (Olympus Corporation).

## Results 

During the study 21 white-eared opossums (*D. albiventris*) were analyzed: nine males and three females. Due to polytrauma, many animals had severe injuries in the abdominal cavity that prevented sex identification, leaving the sex of the remaining animals undetermined. All opossums succumbed to hypovolemic/neurogenic shock resulting from vehicular polytrauma.

**Figure 1:**
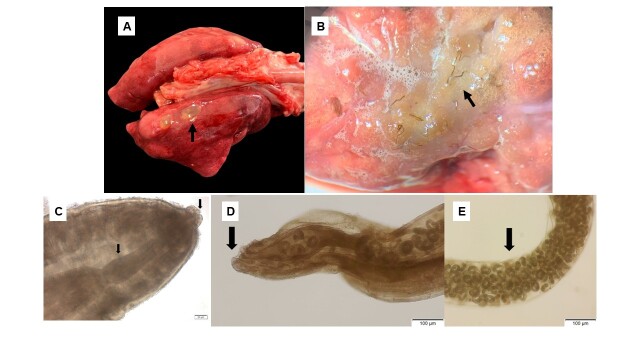
Macroscopic features of white-eared opossum lungs (A and B) and light microscopy features of *Heterostrongylus heterostrongylus* (C to E). (A) Well-defined cystic structures approximately 0.3 cm in diameter on the pleural surface (indicated by arrow). (B) Brown, tapered nematodes within the lung parenchyma (indicated by arrow), accompanied by pulmonary edema. (C) Light microscopy of *H. heterostrongylus*: Female anterior end showing robust trilobed labia and esophagus, each indicated by arrow. (D) Digitiform tail of *H. heterostrongylus* (indicated by arrow). (E) Female posterior end with elliptical and embryonated eggs (indicated by arrow).

Key macroscopic pulmonary findings included marked diffuse hemorrhage in nine animals (9/21), pronounced diffuse edema (7/21), interstitial pneumonia (5/21), congestion (5/21) and lung lobe rupture (3/21). In three individuals, well-defined subpleural nodules and cysts, approximately 0.3 cm in diameter, were observed in the caudal lobes (Figure 1A). In some cases, these nodules appeared linearly arranged. Utilizing a stereomicroscope, brown, tapered nematodes roughly 0.2 cm long were detected interspersed within the lung parenchyma (Figure 1B) (4/21).

**Figure 2:**
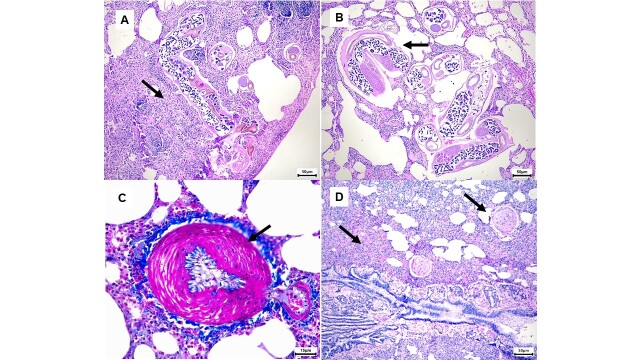
Photomicrographs of *Didelphis albiventris* lungs infected with *Heterostrongylus heterostrongylus:* A. Lung parenchyma exhibiting interstitial pneumonia with adult nematodes in the alveoli. Note the increased cellularity that caused thickening of the alveolar walls (indicated by arrow). Hematoxylin and eosin (HE) staining. Scale bar: 50 µm. B. Adult nematodes with larvae in their reproductive systems alongside some distended and emphysematous alveoli (indicated by arrow). HE staining. Scale bar: 50µm. C. Pulmonary arteriole displaying smooth muscle hypertrophy in the tunica media (indicated by arrow). Masson's trichrome staining. Scale bar: 15 µm. D. Pulmonary vasculature section showing significant thickening of smooth muscles in the pulmonary arteries and arterioles, accompanied by marked inflammatory infiltrate (indicated by arrow). HE staining. Scale bar: 50 µm.

Microscopic examination identified pulmonary nematodes in nine (42.85%, CI_95_%=0.2169, 0.6402) animals, located in the alveoli and bronchioles. Parasite counts in the histological sections ranged from one to 18 individuals per host. Both adult and immature nematodes were seen in transverse, longitudinal and tangential sections. Mature adults exhibited a thin cuticle, coelomic musculature, and a pseudocoelom housing a prominent digestive tract and reproductive organs including an ovary, uterus and larvae at various development stages (Figures 2A and 2B). The immature nematode forms, although similar to the adults, did not exhibit a reproductive tract or larvae in the observed histological sections. The distribution of parasites included the alveolar parenchyma (100%), parenchyma and bronchioles (22%), and, in one case, the parenchyma and vascular lumen (11.1%).

The most common histological alterations in parasitized animals were interstitial pneumonia (9/9) (Figure 2A), hyperplasia of associated lymphoid tissue (BALT) (9/9), pulmonary hemorrhage (9/9), smooth muscle hypertrophy in both large and small vessels (8/9) (Figure 2C), congestion (8/9) and pulmonary edema (6/9). The inflammatory infiltrate was primarily lymphocytic, with total occlusion of vessel lumens by proliferative changes noted in severe cases (Figure 2D). Eosinophilic infiltration associated with parasites was present in six animals, while marked perivascular fibrosis (2/9) and focal mineralization of the lung parenchyma (1/9) were less frequent.

In 12 animals without detectable parasites, the histological analysis showed hypertrophy of the smooth muscular layer of large and small vessels (100%); interstitial pneumonia (5/12, 41.6%), BALT hyperplasia and edema (9/12, 75%), and congestion (7/12, 58.3%) were observed.

Morphological analysis of the nematodes confirmed sexual dimorphism among the adults, with females being larger and more robust than males. The anterior end of the nematodes features two robust trilobed labia and a cephalic collar, which consists of a folded cephalic cuticle (Figure 1C). The female's anterior end displays a claviform esophagus (Figure 1C). The female tail is digitiform (Figure 1D). The visualization technique also revealed a gubernaculum equipped with two spicules. The eggs, elliptical and embryonated, possess a thin hyaline shell, and rhabditoid first-stage larvae were observable under a microscope (Figure 1E). These morphological details confirm the identification of the parasite as *Heterostrongylus heterostrongylus* (Travassos, 1925). No other concomitant parasitic infections were identified in the pulmonary tissues of the animals.

## Discussion

Knowledge about the effects of lung nematode parasitism on the respiratory system of marsupials remains scarce. This issue gains importance as human encroachment on natural habitats increases their proximity to urban areas, coinciding with limited data on helminth fauna, their zoonotic potential, and health impacts on marsupials. In our study, we characterized *H. heterostrongylus* infections in the lungs of *D. albiventris.* This parasite had been previously reported in *D. aurita* (Costa et al., 2016).

Two lungworm species, *H. heterostrongylus* and *D. hayesi*, are known to infect opossums. In Brazil, *H. heterostrongylus* infections in *D. aurita* were noted in the Rio de Janeiro region by Costa (2017). Moreover, *D. hayesi* has been identified in *D. albiventris* with notable prevalence (Antunes, 2005), and lung infections by *D. hayesi* have been recorded in other opossum species, such as *D. virginiana* (Baker et al., 1995; Lamberski et al., 2002, Nichelason et al., 2008; López-Crespo et al., 2017) and *D. marsupialis* (Prestwood et al., 1977).

Anderson et al. (1980) noted that *H. heterostrongylus* and *D. hayesi* have very similar morphologies. However, species differentiation is possible through specific features like the arrangement of the rays and the shape of the tail's terminal portion. Females of *D. hayesi* have a short, rounded tail, while those of *H. heterostrongylus* feature a digitiform tail, as observed in our specimens. The identification of adult parasites in opossum lungs, marked by sexual dimorphism, two robust trilobed labia, a cephalic collar, digitiform-tailed females, males with a gubernaculum and two spicules, and elliptical, embedded eggs, corresponds with the description of *H. heterostrongylus* by Costa et al. (2016).

One limitation of studies involving road-killed animals is the prevalence of trauma-related injuries. In our study, the analysis of macroscopic features was restricted due to circulatory disturbances, such as hemorrhage and pulmonary edema. Nonetheless, we observed multifocal cysts on the pleural surface of caudal lobes in three animals, a condition previously associated with *D. hayesi* infections in opossums (López-Crespo et al., 2017).

Histopathologically differentiating *H. heterostrongylus* from *D. hayesi* in lung tissue is challenging since both species belong to the Angiostrongylidae family (Anderson, 2009) and exhibit similar morphologies (Costa et al., 2016). The observed morphological features in histological samples align with earlier identifications of *H. heterostrongylus* (Costa et al., 2016). Parasite localization corresponded with typical regions for lung parasites. Pulmonary lesions predominantly featured lymphocytic infiltrates in alveolar walls, with fewer extent macrophages and plasma cells. Notably, eosinophils were seen encircling the parasites, a finding consistent with reports for both nematode species infecting opossums (Costa et al., 2016). However, unlike previous findings, no goblet cell hyperplasia or disruption of bronchiolar muscular walls was observed (Costa et al., 2016; López-Crespo et al., 2017). Smooth muscle hypertrophy in the pulmonary vasculature was noted in nearly all cases (20/21), including in animals without parasites. The absence of parasites in certain lung sections does not rule out their presence in other lung areas. Additionally, lower parasitic loads may complicate the visualization of parasites in histopathological sections. It is hypothesized that these pathological changes can result from vasoactive substances released in the affected lung tissue, though the exact pathological mechanisms remain unclear. These challenges underscore the necessity for meticulous sampling and the incorporation of complementary methods to enhance nematode detection in histopathological studies.

Road-killed wild animals provide valuable insights into the health status of wildlife and environmental conditions. Our research revealed that moderate to severe pulmonary changes commonly affect white-eared opossums infected by *H. heterostrongylus*. The predominant findings were interstitial pneumonia and proliferative vascular changes, present in animals with or without pulmonary parasites. The parasite-induced pulmonary lesions ranged from moderate to severe intensity, suggesting an impact on the animals' health. However, trauma-induced lesions were the cause of death in these specimens.

In summary, this study marks the first documentation of *H. heterostrongylus* infection in *D. albiventris*, describing its morphological features observed through light microscopy and detailing the associated pulmonary changes from natural lungworm infections, highlighting the severity of the condition. The natural lifecycle of this parasite remains unknown, which raises concerns about the potential transmission risks to both domestic and wild animals. Given that *D. albiventris* frequently occupies areas at the interface between wild and peri-urban environments – owing to human activity and its adaptable nature- this risk should not be overlooked.
